# Deuterium oxide validation of bioimpedance total body water estimates in Hispanic adults

**DOI:** 10.3389/fnut.2023.1221774

**Published:** 2023-08-24

**Authors:** Grant M. Tinsley, Kyung-Shin Park, Catherine Saenz, Ayush Mehra, Michael R. Esco, Stefan A. Czerwinski, Brett S. Nickerson

**Affiliations:** ^1^Department of Kinesiology & Sport Management, Texas Tech University, Lubbock, TX, United States; ^2^College of Nursing and Health Sciences, Texas A&M International University, Laredo, TX, United States; ^3^Department of Human Science, The Ohio State University, Columbus, OH, United States; ^4^Department of Kinesiology, University of Alabama, Tuscaloosa, AL, United States; ^5^School of Health and Rehabilitation Sciences, The Ohio State University, Columbus, OH, United States

**Keywords:** hydration, BIA, dilution, body composition, fat-free mass, lean mass

## Abstract

**Background:**

To date, body composition assessments in Hispanics, computed via bioimpedance devices, have primarily focused on body fat percent, fat mass, and fat-free mass instead of total body water (TBW). Additionally, virtually no information is available on which type of bioimpedance device is preferred for TBW assessments in Hispanic populations.

**Purpose:**

The purpose of this study was to validate two bioimpedance devices for the estimate of TBW in Hispanics adults when using a criterion deuterium oxide (D_2_O) technique.

**Methods:**

One-hundred thirty individuals (males: *n* = 70; females: *n* = 60) of Hispanic descent had TBW estimated via D_2_O, single-frequency bioimpedance analysis ([SF-BIA] Quantum V, RJL Systems) and bioimpedance spectroscopy ([BIS] SFB7 Impedimed).

**Results:**

The mean values for SF-BIA were significantly lower than D_2_O when evaluating the entire sample (37.4 L and 38.2 L, respectively; *p* < 0.05). In contrast, TBW values were not statistically significant when comparing D_2_O against BIS (38.4 L, *p* > 0.05). Bland–Altman analysis indicated no proportional bias when evaluating the entire sample for SF-BIA or BIS. The standard error of estimate and total error values were ≤ 2.3 L and Lin’s concordance correlation coefficient were ≥ 0.96 for all comparisons.

**Conclusion:**

The SF-BIA and BIS devices evaluated in the current study hold promise for accurate estimation of TBW in Hispanic adults. While both methods demonstrated relatively low errors relative to the D_2_O criterion, BIS exhibited a more consistent performance, particularly at the group level. These findings provide essential information for researchers and clinical nutrition practitioners assessing TBW in Hispanic adults.

## Introduction

Multi-compartment models are highly regarded for body composition assessments due to the ability to account for the aqueous content of fat-free mass (1–3). For example, previous research has shown that fat-free mass hydration varies between 68–81% (4). As a result, total body water (TBW) is an important metric to consider when seeking to quantify body composition in more sophisticated multi-compartment models. The reference standard for laboratory based TBW measurements is deuterium oxide (D_2_O). Although highly desired, administering D_2_O is time-consuming (4–6 h.), expensive, and requires a high level of technical expertise. As a result, the use of D_2_O outside of a well-funded research laboratory is impractical. Consequently, the assessment of TBW via simpler bioimpedance-based methods is a more feasible approach.

Conventional hydration assessments in clinical care have been determined via single-frequency bioimpedance analysis (SF-BIA). This method simply involves the use of gel electrodes and leads that can be connected to a portable bioimpedance device. Once configured, an electrical signal is sent through the body at 50 kHz to measure body impedance (resistance and reactance). Afterwards, total body water (TBW) measurements can be obtained via SF-BIA based on the assumption that the electrical current primarily travels through water. While SF-BIA is convenient for quick hydration assessments, other bioimpedance methods have been developed due to limitations such as the use of regression equations to estimate TBW. Accordingly, sophisticated bioimpedance technology has led to the development of bioimpedance spectroscopy (BIS), which employs Cole modeling (5) and mixture theories (6), instead of equations. This advancement has prompted the idea that BIS is more accurate than SF-BIA. Despite these assertions, a thorough evaluation of SF-BIA and BIS across a single time-point remains elusive, particularly among Hispanic populations.

The assessment of bioimpedance devices in Hispanics has primarily focused on body fat percent, fat mass, and fat-free mass instead of TBW (7, 8). For example, Nickerson and Snarr (9) observed moderate-to-strong proportional bias when comparing a multi-frequency bioimpedance device against dual energy X-ray absorptiometry in Hispanic adults. The error in previous research is likely attributed to deviations in fat-free mass hydration values from the assumed constant of 73.8% that bioimpedance devices employ when estimating body composition (10). For instance, Hispanics fat-free mass hydration values have been found to range from 63.76–79.55% (11). Accordingly, these findings highlight the importance of including a measure of TBW when seeking to estimate body composition in Hispanic adults. However, research is lacking in this area. Additionally, there is no agreement on whether BIS is preferred over SF-BIA for TBW measurements when D_2_O is unavailable in a Hispanic population. Therefore, the purpose of this study was to validate two bioimpedance devices (SF-BIA and BIS) for the estimate of TBW in Hispanics adults when using a criterion D_2_O technique.

## Materials and methods

### Participants

One-hundred thirty individuals (males: *n* = 70; females: *n* = 60) of Hispanic descent had body composition estimated via D_2_O, SF-BIA, and BIS. Recruitment occurred via flyers, word of mouth, and classroom recruitment. Inclusion criteria consisted of Hispanic females and males that were: (1) 18–65 years of age and apparently healthy (i.e., free from orthopedic disorders and who had no known signs or symptoms of cardiovascular, pulmonary, or metabolic diseases), (2) < 350 lbs., and (3) did not have conditions or take medications that may affect body composition. Participants were asked to complete an overnight fasting protocol, which consisted of not eating or drinking 8 h prior to participation and to also avoid exercise 24 h before testing. Prior to testing, participants provided written informed consent and completed a self-reported medical history questionnaire to ensure inclusion criteria were met. This study was conducted according to the guidelines laid down in the Declaration of Helsinki and all procedures involving human subjects were approved by the Institutional Review Board of the host university (IRB # 2016-10-16).

### Procedures

Upon completion of the informed consent and medical history questionnaire, participants’ hydration status was assessed from a urine sample using a handheld refractometer. Urine specific gravity (USG) values <1.029 were required for inclusion in this analysis (12). After assessing hydration, height was measured (to the nearest 0.1 cm) with a stadiometer that has a maximum capacity of 205 cm (SECA 213, Seca Ltd., Hamburg, Germany).

### Deuterium oxide

Criterion TBW was conducted using D_2_O (99.8% 2H, Cambridge Isotope Laboratories, Inc., Andover, MA, United States). Prior to D_2_O ingestion, urine samples were collected from all participants. Each participant was instructed to void their bladders as much as possible. Urine samples obtained during this time point were used for baseline analysis. After voiding the bladder completely, participants ingested ≈ 11 grams of D_2_O along with a 100 mL rinse of deionized water. The exact amount of D_2_O ingested for each participant was recorded. Subjects were then asked to void their bladder at 3.5 h to clear the bladder of any urine that had not been completely diluted. Next, subjects waited another 30 min and were instructed to provide a post-urine sample at 4 h. Participants were asked to remain in the Body Composition Laboratory during the 4 h equilibration period to ensure that eating, physical exertion, and other factors that can impact results did not occur prior to the post-urine sample collection. Urine-diluted D_2_O was analyzed in triplicate using an isotope-ratio mass spectrometer at an independent laboratory (Metabolic Solutions, Inc., Nashua, NH). Isotope abundances in the urine was calculated following the method of Wong et al. (13). Total body water was calculated from the dilution of isotopic water and corrected for the exchange of D_2_O with nonaqueous tissue.

### Single-frequency bioimpedance analysis

Subjects had their TBW measured with SF-BIA (Quantum V, RJL systems, Clinton MI). For SF-BIA testing, the subjects’ right and left shoe and sock were removed, and their arms were placed ≥30° away from the body with legs separated and not touching. Excess hair at electrode sites was removed and the skin was cleaned with alcohol pads and dried prior to electrode placement. Surface electrodes were placed on the right and left wrist beside the ulnar head and on the first joint of the middle finger. Surface electrodes were also placed on the right and left foot beside the medial malleolus and on the base of the second toe. Next, leads were attached to the eight electrodes and a single-frequency (i.e., 50 kHz) whole-body impedance measurement was obtained for each subject to calculate TBW. All TBW measurements were computed using the built-in SF-BIA algorithm.

### Bioimpedance spectroscopy

Subject’s TBW was also determined via BIS (Imp™ SFB7, ImpediMed Limited, Queensland, Australia). Testing occurred immediately after SF-BIA scans. For testing, the subjects’ right shoe and sock remained off and their arms placed ≥30° away from the body with legs separated and not touching. Two single tab electrodes were placed at the distal end of the subject’s (1) right wrist and hand and (2) right ankle and foot, with 5 cm between each set of electrodes in order measure TBW. The BIS device employed 256 frequencies based upon Cole modeling (5) and mixture theories (6) rather than regression equations. Lastly, the TBW measurements were computed using the inherent BIS calculation.

### Statistical analysis

For all analyses, the D_2_O TBW value was considered as the criterion measure, with alternate TBW estimates provided by BIS and SF-BIA. All analyses were performed in the entire sample (*n* = 130), as well as females (*n* = 70) and males (*n* = 60) individually. Traditional null hypothesis significance testing was performed through one-way analysis of variance and follow-up pairwise comparisons with Tukey adjustment. Additionally, equivalence testing was performed with 90% confidence limits for two one-sided *t*-tests (TOST) to assess whether each individual bioimpedance method demonstrated equivalence with D_2_O, using a ± 1-liter equivalence region (14).

Bland–Altman analysis was performed, Bland and Altman (15) including generation of the 95% limits of agreement and linear regression to allow for examination of proportional bias (i.e., a slope differing from 0). Ordinary least squares regression was performed to compare the slope and intercept of the linear relationship observed between methods to the line of identity (i.e., a perfect linear relationship, with an intercept of 0 and slope of 1). Additional validity metrics were also considered. The constant error (CE) was calculated as the mean of the individual differences in TBW between D_2_O and each bioimpedance technique, and total error (TE) was calculated as the root mean square error between D_2_O and each bioimpedance technique. Standard error of the estimate (SEE) was defined as the residual standard error value from ordinary least squares regression. Other values of interest included Lin’s concordance correlation coefficient (CCC), Pearson’s correlation coefficient (*r*), and the coefficient of determination (*R*^2^).

All data analysis was performed using R (v. 4.1.2) and the software packages *afex* (v 1.0–1), *emmeans* (v. 1.7.2), *TOSTER* (v. 0.4.0), and *DescTools* (v. 0.99.44) (16–20). Statistical significance was accepted at *p* < 0.05.

## Results

### All participants

**Figure 1:**
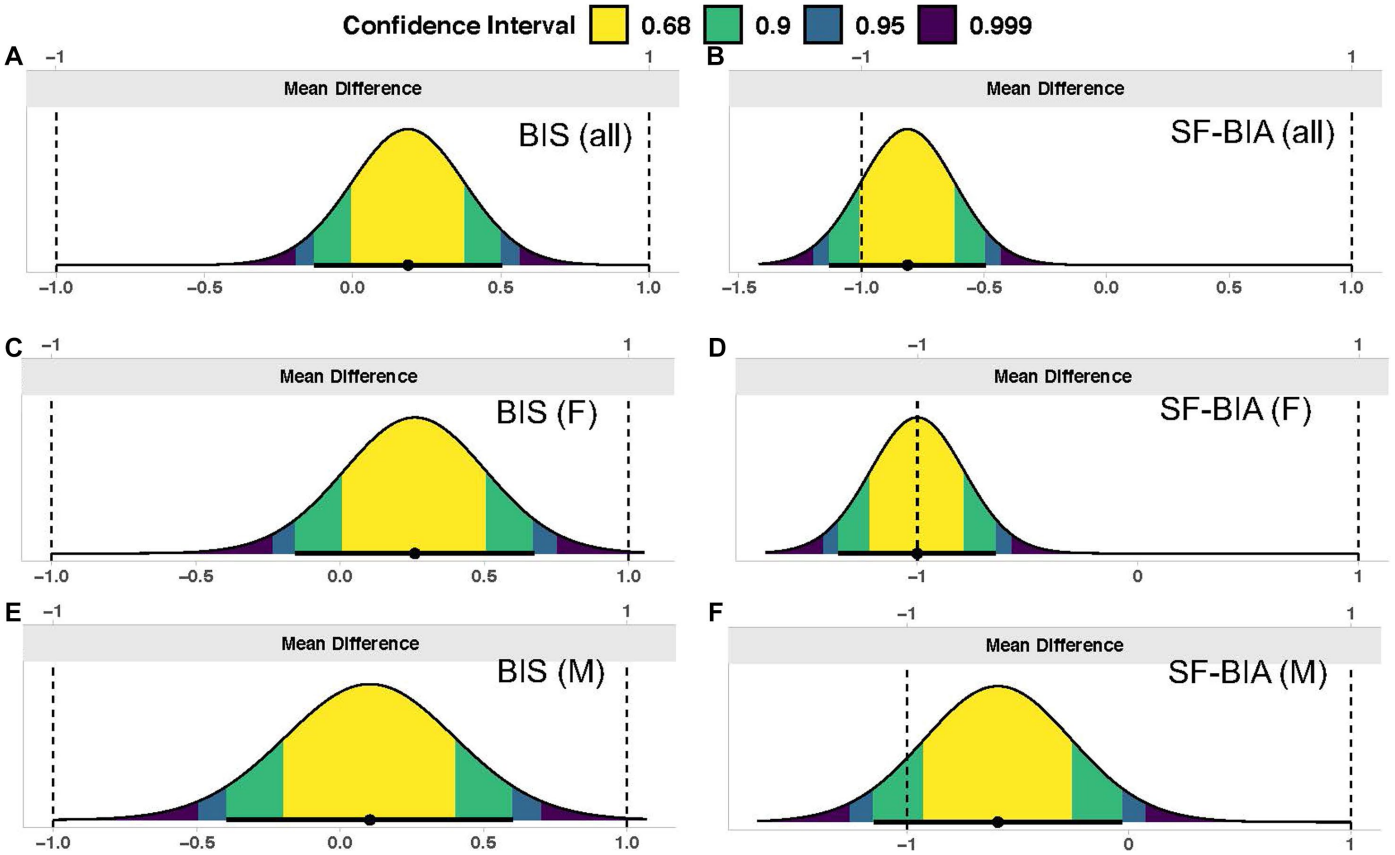
Equivalence testing. In each panel, the distribution of mean differences is displayed, along with confidence intervals. The entirety of a 90% two one-sided *t*-tests (TOST) interval being contained within the specified equivalence region (±1 liter) indicates statistical equivalence between the deuterium oxide criterion and a given bioimpedance technique. Results for the entire sample (*n* = 130) are displayed for **(A)** BIS (ImpediMed SFB7) and **(B)** SF-BIA (RJL Quantum V); results for females only (F; *n* = 70) are displayed for **(C)** BIS and **(D)** SF-BIA; and results for males only (M; *n* = 60) are displayed for **(E)** BIS and **(F)** SF-BIA.

**Figure 2:**
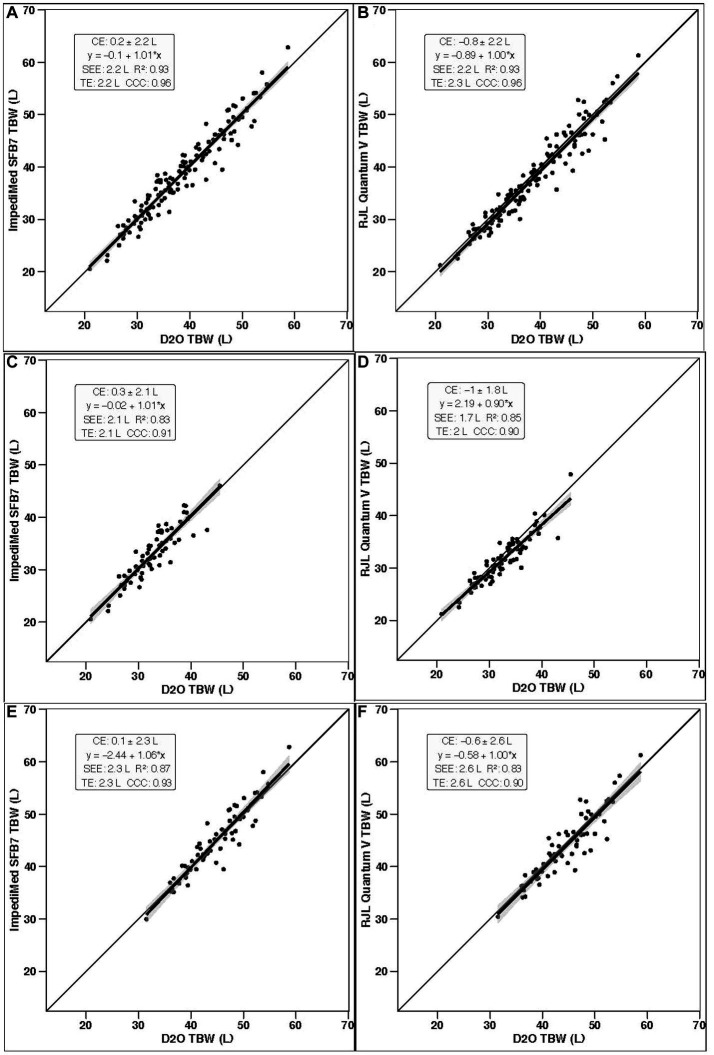
Line of identity comparisons. In each panel, the ordinary least squares regression line indicating the performance of both bioimpedance devices for estimating total body water (TBW) as compared to the line of identity (i.e., perfect agreement with the deuterium oxide criterion) is displayed. The shaded regions indicate the 95% confidence limits for the regression line. Constant error (CE), regression line equations, standard error of the estimate (SEE), coefficient of determination (*R*^2^), total error (TE), and Lin’s concordance correlation coefficient (CCC) are also presented. Results for the entire sample (*n* = 130) are displayed for **(A)** BIS (ImpediMed SFB7) and **(B)** SF-BIA (RJL Quantum V); results for females only (*n* = 70) are displayed for **(C)** BIS and **(D)** SF-BIA; and results for males only (*n* = 60) are displayed for **(E)** BIS and **(F)** SF-BIA.

**Figure 3:**
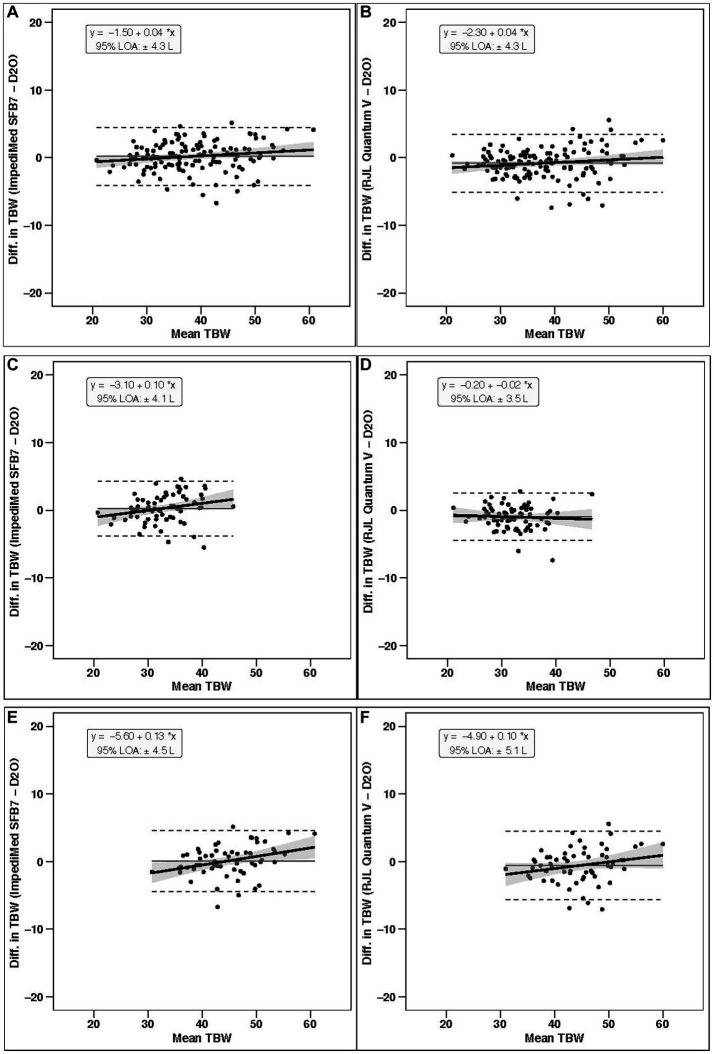
Bland–Altman analysis. In each panel, the relationship between the average of the total body water estimates of a given bioimpedance device and the reference deuterium oxide (D_2_O) method (*x*-axis) and the difference in the estimate from the bioimpedance device minus that of the deuterium method (*y*-axis) is displayed. The linear regression line and associated 95% confidence limits, indicated by the shaded regions, indicate the degree of proportional bias. Horizontal dashed lines indicate the upper and lower limits of agreement (LOA), and the horizontal solid line indicates the constant error between methods. Linear regression equations and 95% LOA values are also displayed. Results for the entire sample (*n* = 130) are displayed for **(A)** BIS (ImpediMed SFB7) and **(B)** SF-BIA (RJL Quantum V); results for females only (*n* = 70) are displayed for **(C)** BIS and **(D)** SF-BIA; and results for males only (*n* = 60) are displayed for **(E)** BIS and **(F)** SF-BIA.

In the entire sample (*n* = 130; Table 1), TBW estimates significantly differed by method (*p* < 0.001 via one-way ANOVA). Follow up pairwise comparisons indicated differences when comparing SF-BIA against D_2_O (*p* = 0.0003) and BIS (*p* < 0.0001). No differences were found for other comparisons (*p* > 0.48 for all). Additionally, BIS (*p* < 0.0001, Figure 1A), but not SF-BIA (*p* = 0.16, Figure 1B) demonstrated equivalence with D_2_O for TBW estimates. For BIS, the slope and intercept of the best fit line did not significantly differ from the line of identity (Figure 2A); additionally, Bland–Altman analysis indicated no proportional bias (Figure 3A). For SF-BIA, the slope and intercept of the best fit line did not significantly differ from the line of identity (Figure 2B); additionally, Bland–Altman analysis indicated no proportional bias (Figure 3B). CE values were < 1 L for both methods, with CCC values ≥0.96, and SEE and TE values ≤2.3 L.

**Table 1:** Participant characteristics.

	All (*n* = 130)		Females (*n* = 70)		Males (*n* = 60)	
	Mean	SD	Mean	SD	Mean	SD
Height (cm)	166.6	8.8	160.7	5.8	173.4	6.2
Weight (kg)	78.2	17.8	72.1	16.5	85.4	16.7
BMI (kg/m^2^)	28.1	5.8	27.9	6.3	28.3	5.1
Age (y)	29.2	11.3	30.0	11.2	28.2	11.5
D20 (g)	10.9	0.2	10.9	0.2	10.9	0.1
Waist (cm)	91.0	15.5	88.5	16.5	93.9	13.9
Hip (cm)	104.7	10.8	105.8	11.8	103.6	9.3
BIS TBW (L)	38.4	8.3	32.9	5.1	44.8	6.5
SFBIA TBW (L)	37.4	8.2	31.6	4.5	44.1	6.3
D_2_O TBW (L)	38.2	7.9	32.6	4.6	44.7	5.7

D_2_O, deuterium oxide; BIS, bioimpedance spectroscopy; SF-BIA, single-frequency bioelectrical impedance analysis; TBW, total body water.

### Females

In females (*n* = 70), TBW estimates significantly differed by method (*p* < 0.001 via one-way ANOVA). Follow up pairwise comparisons indicated differences when comparing SF-BIA against D_2_O (*p* = 0.0001) and BIS (*p* < 0.0001). No differences were found for other comparisons (*p* > 0.19 for all). Additionally, BIS (*p* = 0.002, Figure 1C), but not SF-BIA (*p* = 0.50, Figure 1D) demonstrated equivalence with D_2_O for TBW estimates. For BIS, the slope and intercept of the best fit line did not significantly differ from the line of identity (Figure 2C); however, Bland–Altman analysis indicated slight proportional bias (Figure 3C). For SF-BIA, the intercept of the best fit line did not significantly differ from the line of identity, but the slope differed from 1 (Figure 2D); however, Bland–Altman analysis indicated no proportional bias (Figure 3D). CE values were ≤ 1 L for both methods, with CCC values ≥0.90, and SEE and TE values ≤2.1%.

### Males

In males (*n* = 60), TBW estimates did not significantly differ by method (*p* = 0.10 via one-way ANOVA). BIS (*p* = 0.002, Figure 1E), but not SF-BIA (*p* = 0.11, Figure 1F) demonstrated equivalence with D_2_O for TBW estimates. For BIS, the slope and intercept of the best fit line did not significantly differ from the line of identity (Figure 2E); however, Bland–Altman analysis indicated slight proportional bias (Figure 3E). For SF-BIA, the slope and intercept of the best fit line did not significantly differ from the line of identity (Figure 2F); additionally, Bland–Altman analysis indicated no proportional bias (Figure 3F). CE values were ≤ 0.6 L for both methods, with CCC values ≥0.90, and SEE and TE values ≤2.6%.

## Discussion

A greater proportion of Hispanic adults exhibit overweight or obesity as compared to those who are non-Hispanic (82.0% in Hispanic vs. 49.6 to 75.7% in non-Hispanic White, Black, and Asian adults, based on 2015–2018 data from the United States) (21). However, due to limitations of body mass index for evaluation of adiposity (22), consideration of body composition characteristics is warranted. While bioimpedance is a convenient option for body composition monitoring, it has been noted that the validity of bioimpedance in Hispanic populations is yet to be thoroughly evaluated, with most existing comparisons utilizing non-criterion comparison methods (23). As bioimpedance body composition applications typically rely on the estimation of TBW followed by subsequent prediction of body fat and fat-free mass (24), examining the validity of bioimpedance-based TBW estimates in Hispanics is an essential first step to establishing bioimpedance as valid assessment method in this population.

In the present analysis, the validity of default TBW estimates from two distinct, commercially available bioimpedance technologies was examined in Hispanic adults. The primary findings were: (1) in the entire sample, BIS demonstrated the best performance for TBW estimation, as indicated by statistical equivalence and no significant difference from D_2_O, no deviation from the line of identity, and no proportional bias; when separated by sex, these features were also seen, except for the presence of slight proportional bias (slopes of 0.10 to 0.13) and (2) in all analyses, SF-BIA failed to demonstrate statistical equivalence and differed significantly from D_2_O, but best fit lines typically did not deviate from the line of identity; additionally, proportional bias was not observed in any analysis. When examining additional validity metrics, both bioimpedance methods demonstrated relatively low error relative to D_2_O (CE < 1 L, CCC ≥ 0.96, SEE and TE ≤ 2.3 L, and LOA ≤ 4.3 L in the entire sample). Collectively, these results suggest that both bioimpedance devices analyzed in the current study hold promise for accurately estimating TBW in groups of Hispanic adults, but that BIS demonstrated the best overall performance in the present analysis.

Although Hispanics have traditionally been underrepresented in body composition methodology research, select works have reported the agreement between body composition technologies or described body composition characteristics of Hispanic populations (9, 11, 23, 25). Several of these studies have demonstrated sex differences in the performance of bioimpedance technologies in Hispanic populations, with greater errors in females as compared to males (9, 26). These findings provided the rationale for examining sex differences in the present analysis; however, with minor exception, performance of the selected bioimpedance technologies were similar in males and females in the present study. This is notable as prior work demonstrating sex differences compared bioimpedance-based body composition estimates to those derived from dual-energy X-ray absorptiometry (9, 26), which is a limitation compared to multi-compartment models including TBW estimates (3, 4, 27). In contrast, the present study employed a criterion estimate of TBW, indicating the previously documented sex differences could be due to the comparison method used or sex differences in fat-free mass properties that could have introduced errors when predicting fat-free mass from TBW (11, 25). While outside the scope of the present work, deviation from assumed values of certain body components, such as fat-free mass hydration, in specific racial or ethnic groups necessitates race-specific evaluation of commonly used assessment methods (7–9, 11, 25). Finally, characteristics of the Hispanic populations in existing research have varied, with some works including Hispanic individuals of Caribbean origin (26) and others, including the present study, predominantly evaluating Mexican-Americans (9).

Although the present results support the use of select bioimpedance technologies for TBW estimation in Hispanic adults, these conclusions should not be indiscriminately applied to all bioimpedance technologies due to differences in physical characteristics and TBW estimation algorithms (28, 29). For instance, the posture used during bioimpedance assessments (supine vs. standing) has been demonstrated to influence raw bioelectrical properties (28, 30). Notably, the current study used two bioimpedance devices that require lying supine during testing. Although SF-BIA and BIS demonstrated strong group-level performance relative to D_2_O in the present analysis, it is unknown whether these findings extend to bioimpedance devices that require standing during testing. Accordingly, the accuracy of octopolar bioimpedance devices that require standing should be further evaluated in a Hispanic population for the estimation of TBW.

Strengths of the present investigation include the use of the criterion D_2_O method for TBW estimation, the use of two commercially available bioimpedance analyzers representing a range of specific technologies (BIS and SF-BIA), and the recruitment of an understudied population. Limitations include a lack of direct comparison with other racial/ethnic groups and the use of a single testing site, which could potentially limit the generalizability of these findings to some contexts. The focus of the present analysis was the validation of default TBW estimates produced by two commercially available bioimpedance technologies, and the strong performance may indicate a lack of need for Hispanic-specific TBW prediction equations. Nonetheless, additional exploration of this question in additional samples is warranted before definitive conclusions should be established.

In conclusion, two commercially available bioimpedance technologies hold promise for accurate estimation of TBW in Hispanic adults. While both methods demonstrated relatively low errors relative to the D_2_O criterion, BIS exhibited better performance than SF-BIA, particularly at the group level. Nonetheless, SF-BIA performed well as assessed by several metrics, such as demonstrating a lack of proportional bias, systematic differences in TBW estimates were observed. However, both technologies demonstrated relatively low error that may be acceptable in some settings. These findings provide essential information for researchers and clinical nutrition practitioners assessing TBW in Hispanic adults.

## Data availability statement

The raw data supporting the conclusions of this article will be made available by the authors, without undue reservation.

## Ethics statement

The studies involving humans were approved by Texas A&M International University. The studies were conducted in accordance with the local legislation and institutional requirements. The participants provided their written informed consent to participate in this study.

## Author contributions

BN designed the study and collected the data for analysis. GT analyzed and interpreted the data. BN and GT wrote the manuscript with input from CS, K-SP, ME, AM, and SC. All authors contributed to the article and approved the submitted version.

## Funding

Research reported in this publication was supported by the National Institute of General Medical Sciences of the National Institutes of Health under Award Number SC1GM135099. The content is solely the responsibility of the authors and does not necessarily represent the official views of the National Institutes of Health.

## Conflict of interest

The authors declare that the research was conducted in the absence of any commercial or financial relationships that could be construed as a potential conflict of interest.

## Publisher’s note

All claims expressed in this article are solely those of the authors and do not necessarily represent those of their affiliated organizations, or those of the publisher, the editors and the reviewers. Any product that may be evaluated in this article, or claim that may be made by its manufacturer, is not guaranteed or endorsed by the publisher.
